# Conformational analysis of a synthetic fish kisspeptin 1 peptide in membrane mimicking environments

**DOI:** 10.1371/journal.pone.0185892

**Published:** 2017-10-04

**Authors:** Dimpal Thakuria, Neetu Shahi, Atul Kumar Singh, Victoria Chanu Khangembam, Arvind Kumar Singh, Satish Kumar

**Affiliations:** 1 ICAR-Directorate of Coldwater Fisheries Research, Bhimtal, Nainital, Uttarakhand, India; 2 ICAR-Indian Veterinary Research Institute, Izatnagar, Bareilly, Uttarpradesh, India; Universite de Rouen, FRANCE

## Abstract

Kisspeptin 1 is a neuropeptide hormone of the RFamide family, which act as an upstream regulator of brain-pituitary-gonad (BPG) axis in most vertebrates including teleosts. In the present study, a 16 amino acid long putative mature bioactive peptide (kiss 1) from preprokisspeptin 1 of golden mahseer, *Tor putitora* (Hamilton, 1822), was synthesized and characterized using an integrated (experimental and *in silico*) approach. The far-UV circular dichroism (CD) spectrum of this peptide was evaluated both in aqueous and membrane mimicking solvents (TFE, HFIP and Dioxane). The results indicate that kiss 1 peptide adopted helical, turn and β conformations in membrane like environments. The near-UV CD spectroscopy was also carried out to examine the tertiary packing around aromatic residues of kiss 1 peptide and the peptide-membrane complex. The kiss 1 peptide exhibited little signal in water, but a prominent negative band was observed at around 275 nm when membrane mimetic solution was added. The observed ordered conformations of kiss 1 peptide in the different solvents indicated its potential biological activity which could enhance the secretion of gonadotropin-releasing hormone (GnRH) at BPG axis. The conformational information generated from the present study reinforces the application prospects of bioactive synthetic peptide analogs of kisspeptin 1 in improving the reproductive performances of important cultivable fish species.

## Introduction

Kisspeptin 1 (kiss 1), a neuropeptide encoded by *kiss1* gene was first shown to play a role in the reproduction, by the discovery that dysfunction of the GPR54/kiss-1 receptor (kiss1R) causes idiopathic hypogonadotropin hypogonadism (iHH) in some patients [1,2]. In brain, the kisspeptin neurons are mainly present in the arcuate and anteroventral periventricular nucleus of hypothalamic region, and projecting into the preoptic area where kiss1R (GPR54) transcripts are colocalized in gonadotropin-releasing hormone (GnRH) neurons [3–8]. Functionally, agonistic and receptor binding abilities of N-terminal truncated kisspeptin-54 peptides have been analyzed by calcium mobilization and competitive binding assays using CHO cells expressing human kiss1R [9] and revealed that C-terminal 10-amino acid peptide, kisspeptin-10 (Kp-10), has 3-to10-fold more potent agonistic and receptor binding activities than the kisspeptin-54 for kiss1R. In fact, it is reported that the modified Kp-10 analog, [dY]^1^ Kp-10, exhibits an *in vivo* bioactivity even higher than the endogenous peptide, Kp-10 [10].

Further, based on the analysis of receptor binding and activity of a number of human Kp-10 analogs, Roseweir et al. [11] emphasized the importance of the five C-terminal amino acids in receptor activation. Specifically, they found that amino acid substitutions at positions 1, 5, and 8 resulted in a high-affinity kiss1R antagonist, suggesting the importance of these positions for the bioactivity of Kp-10. Similarly, in a decapeptide analog of kisspeptin (45–54) developed by substitutions with specific amino acids at D-Tyr^45^, D-Trp^47^, azaGly^51^ and Arg (Me)^53^, Asami et al. [12] found that replacement of amino acid at positions 45–47 or N-terminal truncation of one amino acid (nonapeptide, kiss 1–305) substantially improves the receptor agonistic activity and serum stability. Subsequent to their study, pharmacological profiling of two other nonapeptide Kp analogs TAK-448 and TAK-683, revealed high receptor-binding affinity and fully potent agonistic activity for rat kiss1R, comparable to Kp-10 [13].

Unlike mammals, long kiss peptides are reported to be more potent activators of kisspeptin receptors in several fish species such as zebrafish (*Danio rerio*) [14], striped bass (*Morone saxatilis*) [15], chub mackerel (*Scomber japonicus*) [16], medaka (*Oryzias latipes*) [17] and, European sea bass (*Dicentrarchus labrax*) [18]. Moreover, majority of the fish kisspeptin 1 have -RY-NH_2_ motif at C-terminal as compared to -RF-NH_2_ motif reported in most mammals [14]. Among teleosts, GPR54 receptor in GnRH neurons was first identified in a cichlid fish, tilapia [19]. Following this, kiss 1 and GPR54 systems have been identified in several species of teleosts [20,21]. GnRH, the downstream target of kisspeptin is known to play a significant role in stimulating the synthesis and release of the gonadotropins (GTH) i.e. follicle-stimulating hormone (FSH) and luteinizing hormone (LH), which in turn act on the gonads to stimulate the release of sex steroids that induce gonadal maturation [22]. Therefore, the functional role of kisspeptin as an upstream regulator of puberty and seasonal reproduction in various fishes is assuming greater significance [23].

Aquaculture production has been enormously benefitted by the ability to stimulate spawning as and when desired, even beyond the natural breeding season of a particular species, by hormonal manipulation of gonadal maturation using exogenous LH or GnRH agonists [24]. Recently, synthetic kisspeptin administration was found promising in hypothalamic GnRH stimulation and induction of gonadal maturity in various fish species viz. European sea bass [25], zebrafish [26], goldfish (*Carassius auratus*) [27] and yellow tail kingfish (*Seriola lalandi*) [28]. However, there is no information on the solution conformation of fish kisspeptin 1 peptide in its native membrane environment, which is critical to the development of potent synthetic kisspeptin peptide analogs. Considering the fact that the functional activity and therapeutic potential of any peptide is dependent on its ability to retain its native conformation in biological environment, the present study was undertaken to analyze the conformation of a 16 amino acid long putative mature bioactive peptide from preprokisspeptin 1 of a fish in various membrane mimicking environments by circular dichroism (CD) spectroscopy and *in-silico* analysis.

## Materials and methods

### Materials

The solvents used for peptide synthesis were mostly of HPLC grade and dry in nature. The Fmoc protected amino acids, rink amide 4-methylbenzhydrylamine (MBHA) resin, 1-hydroxybenzotriazole (HoBt), *O*-(Benzotriazol-1-yl)-*N*,*N*,*N′*,*N′*-tetramethyluronium hexafluorophosphate (HBTU), diethyl ether, HPLC water were obtained from Merck (India and Germany) and GL Biochem (Shanghai, China). N, N-dimethylformamide (DMF), dichloromethane (DCM), dioxane, piperidine, acetonitrile (ACN), acetic anhydride, trifluoroacetic acid (TFA) and methanol were from SD Fine chemicals (India). Trifluoroethanol (TFE), hexafluoroisopropanol (HFIP) and diisopropyl ethylamine (DIEA) were from HiMedia (India).

### Peptide synthesis and purification

The solid phase synthesis of the kiss 1 peptide of golden mahseer was carried out on 4-methylbenzhydrylamine (MBHA) rink amide resin (0.65 mmol.g^-1^) with standard methodology using Fmoc-chemistry [29]. The purpose of using rink amide resin was to incorporate amide group to the C-terminus of the peptide as amide group is crucial for its biological activity [30]. In brief, the resin was allowed to swell in N, N-dimethylformamide (DMF) at room temperature for 2 h in a peptide synthesis vessel. The Fmoc protecting group was removed from the resin using 20% piperidine in DMF for 30 min and the resin was washed three times each with DMF and Dichloromethane (DCM). Fmoc-protected amino acid (3 equivalents relative to resin loading), 1-hydroxybenzotriazole (HOBt) (3 equivalents), *O*-(Benzotriazol-1-yl)-*N*,*N*,*N′*,*N′*-tetramethyluronium hexafluorophosphate (HBTU) (2.9 equivalents) and diisopropyl ethylamine (DIEA) (6 equivalents) were dissolved in dry DMF. The solution was mixed with the resin and then left at room temperature for 2.5 h with continuous shaking at moderate speed. The coupling efficiency of the first amino acid was monitored by Kaiser test. The free reactive groups on the resin were capped by the treatment of the resin with a solution of DMF / DIEA / acetic anhydride (193:6:1, v/v/v) to prevent the formation of truncated products and the same was followed for every coupling cycle of new amino acid. After this, the resin was washed three times each with DMF and DCM respectively. This process was repeated until the desired length of the peptide was obtained. Following side chain protected amino acids were used for synthesis of kiss 1 peptide, Asparagine, Glutamine, Serine: trt; Arginine: Pbf; Tyrosine: tBu.

After completion of the whole coupling cycle, the resin was washed three times each with DMF, DCM and methanol and dried overnight in a desiccator. The dry resin (50 mg) was transferred to clean 2.0 mL tubes and a 500 μL solution of trifluoroacetic acid (TFA): phenol: thioanisole: ethanedithiol (EDT): water (82.5:5:5:2.5:5, v/v/v/v/v) (cleavage mixtures) was added. The mixture was allowed to stir at room temperature for 3 h. The mixture was filtered and the resin was rinsed thrice with TFA cleavage solution and the filtrates were pooled together. The filtrate was washed with cooled diethyl ether that allowed the crude peptide to precipitate and the solution was centrifuged at 5000 rpm for 10 min to form a pellet of crude peptide. Ether was decanted, and the crude peptide was dried overnight in a vacuum desiccator.

The crude peptide was purified by reversed phase-high performance liquid chromatography (RP-HPLC) on a reverse phase C-18 column using water (A)/acetonitrile (B) gradient containing 0.1% TFA. Purification was carried out by injecting 200 μL of a solution of kiss 1 peptide (10 mg mL^-1^) at a time into a C-18 semi-prep scale HPLC column (7 × 300 mm; 10 μ particle size). The RP-HPLC experiment was performed with gradient conditions: initial fixed composition 1% B to 100% B over 35 min and brought down to 1% within 2 min, and held for 3 min. Flow rate was 2.0 mL min^-1^. The total time of the gradient run was 40 min. Repeated runs were carried out following the same methodology to get enough purified peptide for further studies. Then, from the pure eluted fraction, the organic solvent was evaporated and finally lyophilized to yield the purified peptide. The purity of the collected fraction was further analyzed on RP-HPLC using the analytical C-18 column (4 × 150 mm; 5 μ particle size) employing same gradient as semi-preparative RP-HPLC. Mass of the peptide was confirmed by matrix-assisted laser desorption/ionization-time of flight-mass spectrometry (MALDI-TOF-MS) from Sandor Life Sciences, India. The matrix used for molecular weight analysis is sinapic acid (Sigma). For Calibration, protein calibration standard (ProtMix, Bruker Daltonics) was used. The instrument used was MALDI-TOF/TOF MS (Bruker Daltonics ULTRAFLEX III) and was run in positive mode.

### *In silico* structural analysis

Kiss 1 peptide sequence was analysed by online available secondary structure predictive methods, PSIPRED [31] and GOR4 [32]. Three-dimensional (3D) structure of the peptide was created using PEP-FOLD tool [33] and visualized by Jmol.

### Circular Dichroism Spectroscopy

The far-UV circular dichroism (CD) spectroscopy of kiss 1 peptide (0.1 mg mL^-1^) was carried out using a Jasco J-810 CD spectropolarimeter (Jasco Corp., Japan). Spectra were recorded in the far-UV region (190–250 nm) with 1 mm path length, 0.1 nm step resolution, 100 nm sec^-1^ speed, 1 s response time, and 1 nm bandwidth. The CD contributions of water/solvents were subtracted from each spectrum. Each spectrum was recorded as an average of four scans with continuous mode. The mean residue ellipticity [θ] (given in deg cm^2^ dmol^-1^) was calculated as [θ] = 100ψ/c.l, where ψ is the observed ellipticity in millidegree, c is the concentration of the sample in mol litre^-1^, and l is the optical path length of the cell in cm. The CD spectropolarimeter was calibrated using the standard solution of ammonium d-10-camphor sulfonate. The computer simulation of CD spectra was used to provide the quantitative estimation of different secondary structures of the kiss 1 peptide in solution when analysed using secondary structure estimation software (Spectra Manager). The CD spectra were recorded in HPLC grade water and trifluoroethanol (TFE) (45 and 90%, v/v), hexafluoroisopropanol (HFIP) (90%, v/v) and dioxane (90%, v/v) to mimic the extracellular matrix and membrane mimicking environments [34,35]. The near-UV CD (255–320 nm) spectra for kiss 1 peptide (1 mg mL^-1^) were also recorded in water, TFE and dioxane solvents using 10 mm cuvette for tyrosine side chain residues.

### Fluorescence spectroscopy

The intrinsic fluorescence emission spectra of kiss 1 peptide were measured in the presence of water and TFE (90%, v/v). Measurements were made using a fluorescence spectrophotometer (LS-55 Perkin Elmer, USA) with excitation at 274 nm in a 1 cm path length quartz cuvette. Emission spectra were recorded over the wavelength range from 290 to 450 nm.

## Results

### Synthesis and purification of peptide

**Fig 1:**
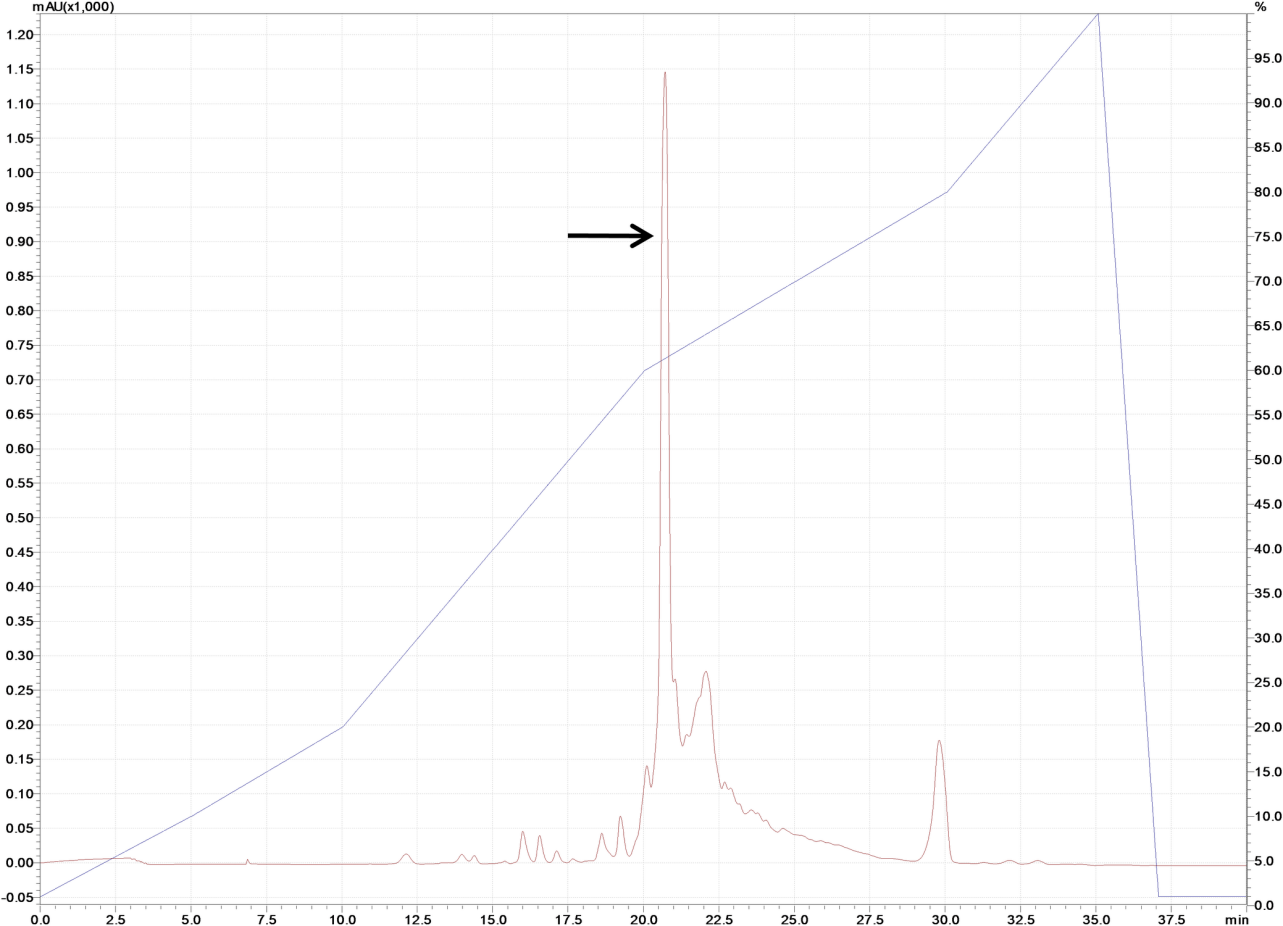
Semi-preparative RP-HPLC chromatogram of kiss 1 peptide at 280 nm using C-18 column. Desired peak is marked by arrow. Retention time is 20.73 min.

**Fig 2:**
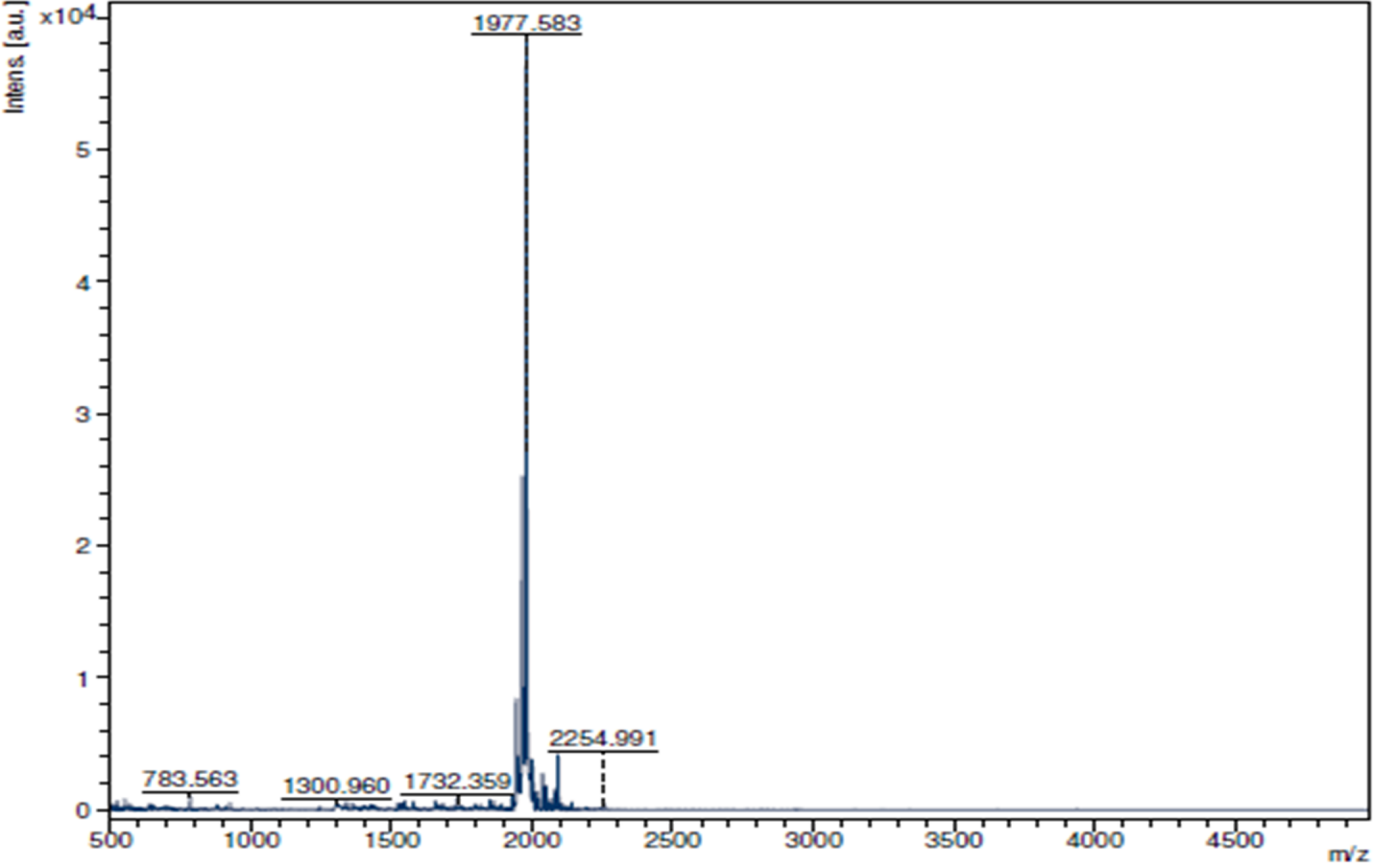
MALDI-TOF mass spectrum of kiss 1 peptide. MS calculated: 1977.19 Dalton, found: 1977p.58 Dalton.

A 16 amino acid long putative mature bioactive peptide, kiss 1 (RQNVAYYNLNSFGLRY-NH_2_) from preprokisspeptin was chosen based on deduced amino acid sequence (NCBI GenBank protein id = "AJT39600.1") of golden mahseer (*Tor putitora*) kisspeptin 1 cDNA (Accession no. KP710729). The peptide was synthesized on an MBHA rink amide resin using Fmoc chemistry. The sample was eluted as a single major peak with retention time (RT) 20.73 min (Fig 1) after optimizing the water/acetonitrile gradient containing 0.1% TFA and the fraction was collected manually. The molecular mass of purified kiss 1 peptide was determined by MALDI-TOF-MS (Fig 2). The detected mass (1977.58 Dalton) of the peptide was found to be consistent with theoretical mass (1977.19 Dalton). The physicochemical properties of the kiss 1 peptide are provided in Table 1.

**Table 1:** Physicochemical properties of kiss 1 peptide.

Number of residues	16
Molecular weight	1977.19
Iso-electric point	pH 10.4
Net charge at pH 7	3
Extinction coefficient	3840 M^-1^cm^-1^

### *In silico* analysis

**Fig 3:**
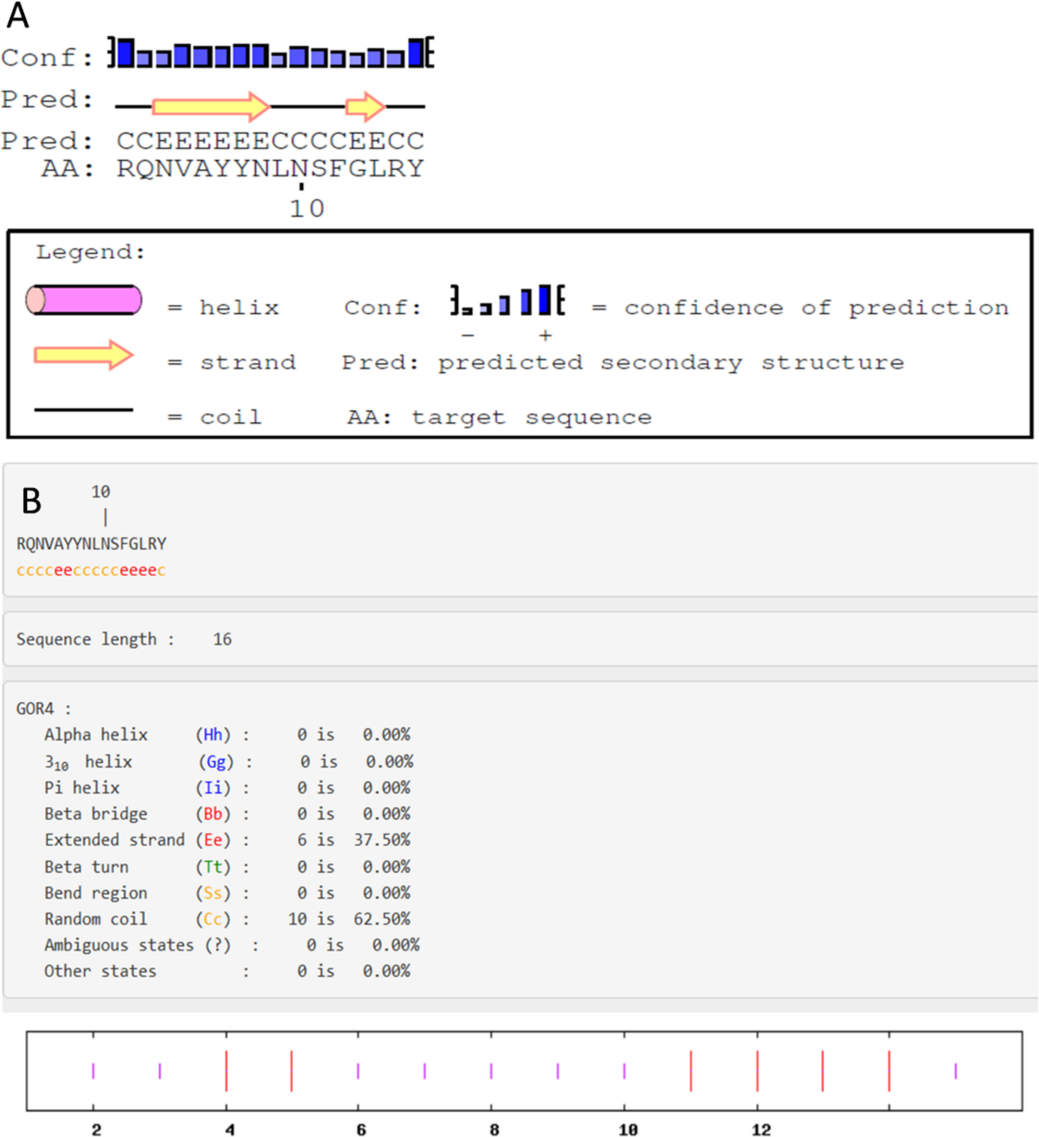
Secondary structure analysis of kiss 1 peptide using online PSI-PRED (A) and GOR4 (B) tools.

**Fig 4:**
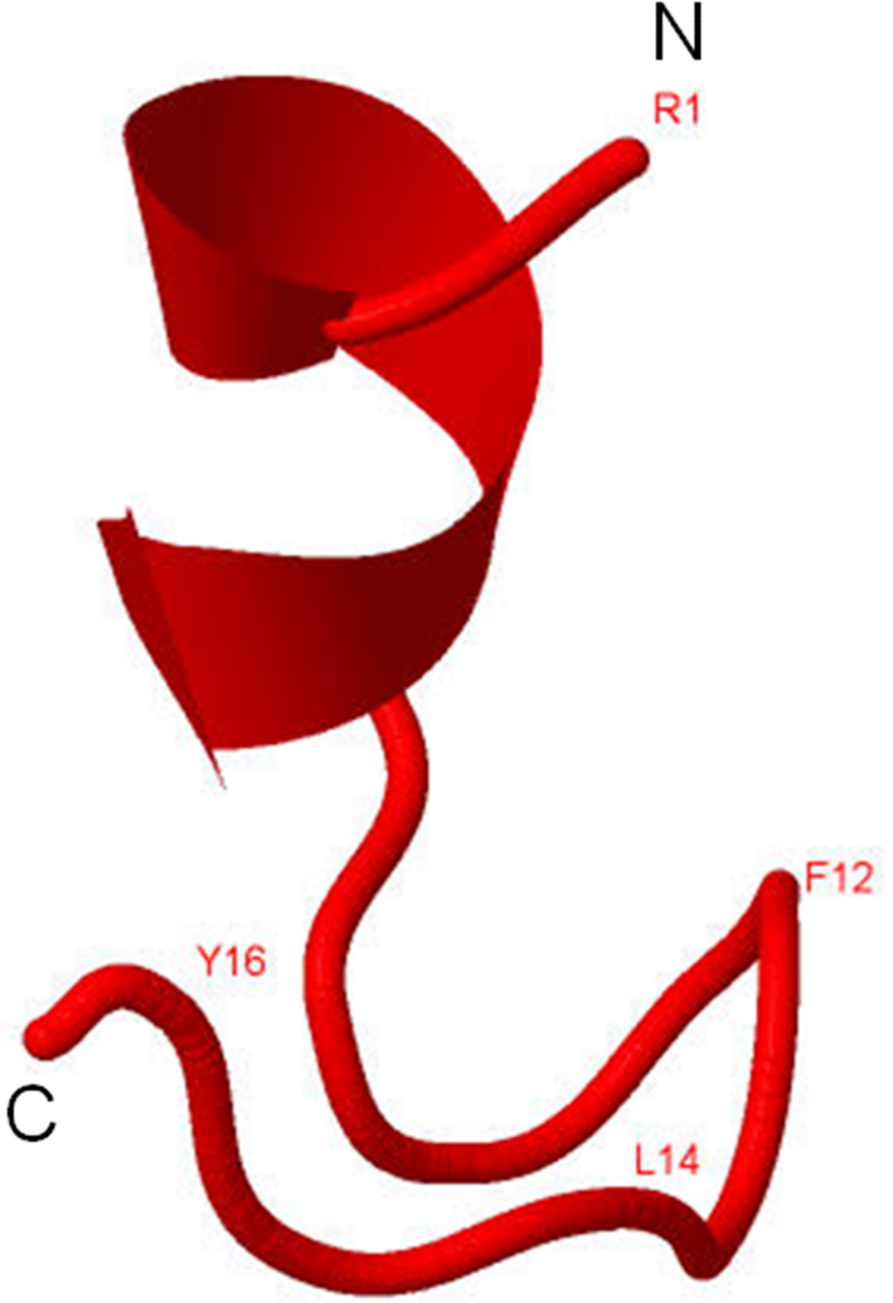
Three dimensional structure analysis of kiss 1 peptide using PEP-FOLD and visualized by Jmol.

Analysis of secondary structure using PSI-PRED and GOR4 showed the presence of β and random coil conformations (Fig 3). In addition, three dimensional (3D) structure of kiss 1 peptide was generated using PEP-FOLD (Fig 4). The predicted 3D structure contains well defined helical and β segments at N- and C- terminal respectively. The predicted structure was further corroborated with CD data.

### Circular Dichroism Spectroscopy

**Fig 5:**
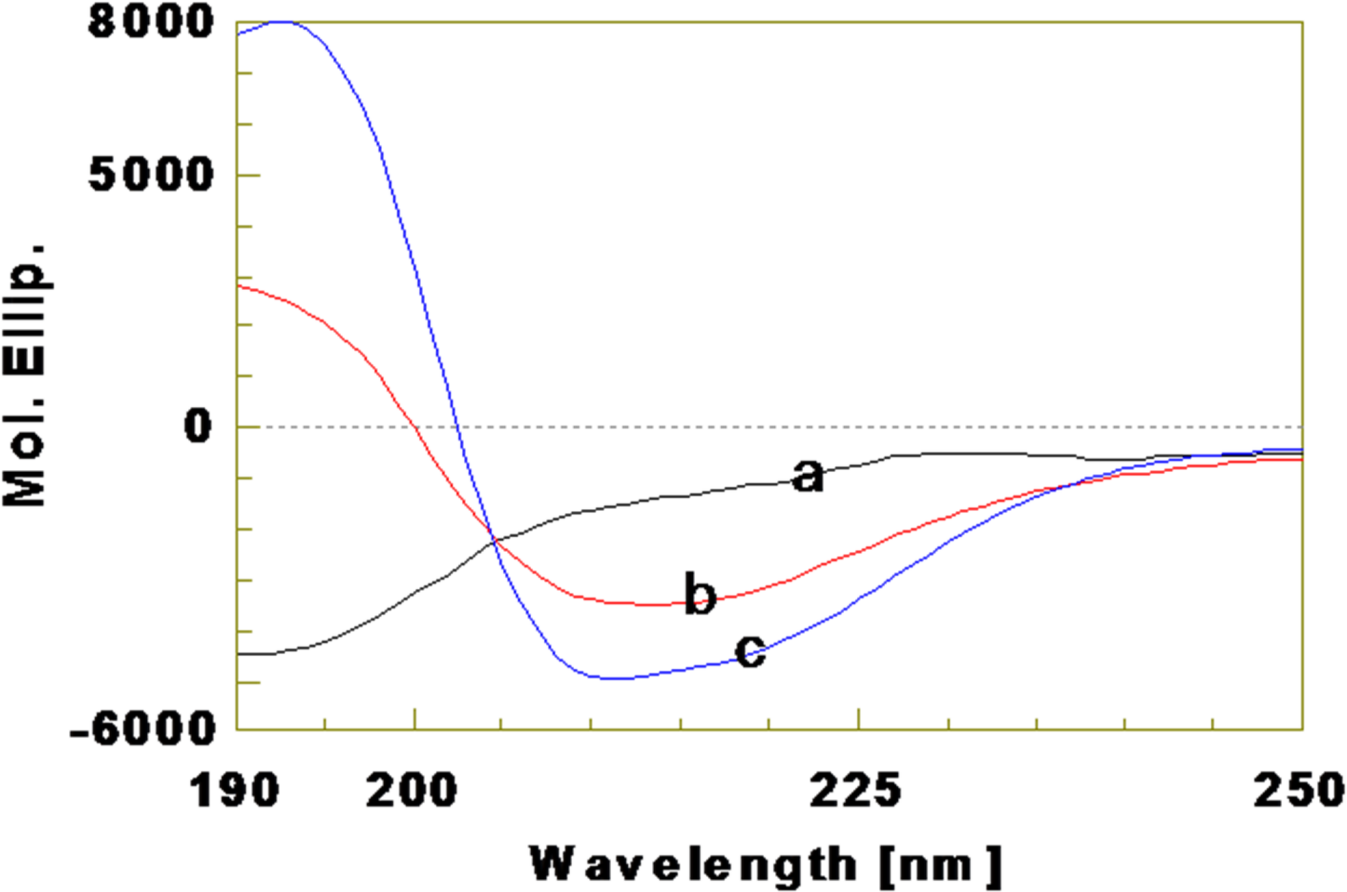
Far-UV CD spectra of kiss 1 peptide in water and TFE. (a) CD spectrum in water. (b) CD spectrum in 45% TFE. (c) CD spectrum in 90% TFE.

**Fig 6:**
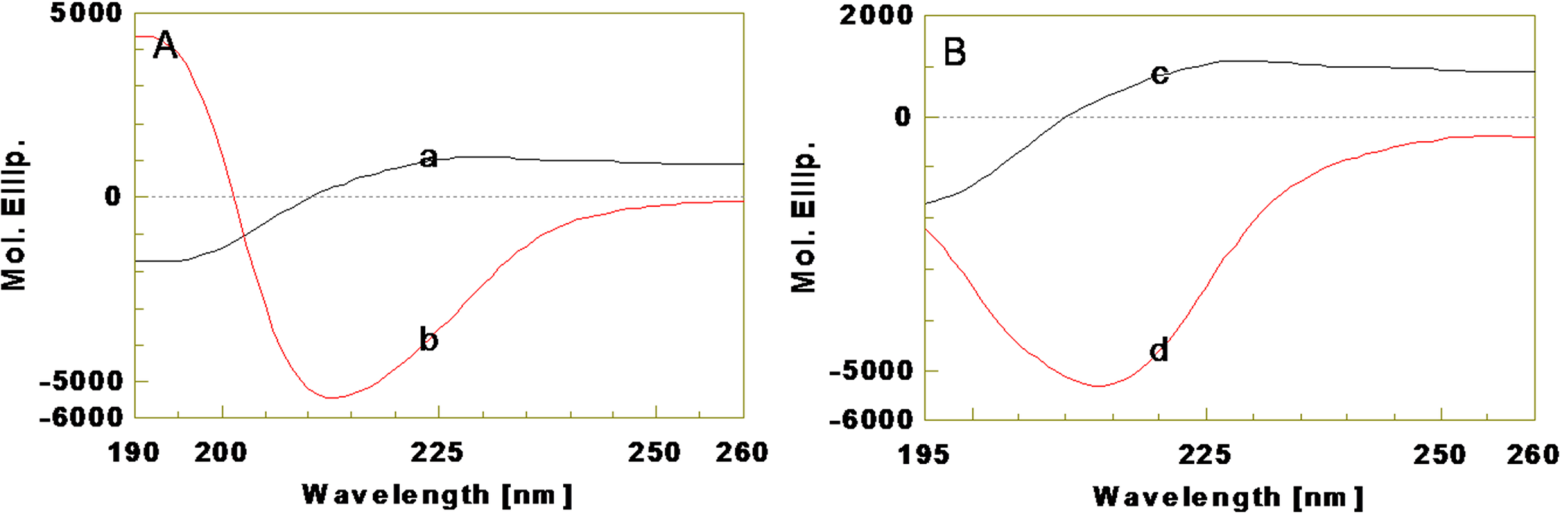
Far-UV CD spectra of kiss 1 peptide in water, HFIP and dioxane. A: (a) CD spectrum in water. (b) CD spectrum in 90% HFIP. B: (c) CD spectrum in water. (d) CD spectrum in 90% dioxane.

The secondary structures of kiss 1 peptide were analyzed using far-UV CD spectroscopy (Figs 5 and 6). CD spectra of kiss 1 peptide were recorded in water and low polarity solvents viz. TFE (45 and 90%, v/v), HFIP (90%, v/v) and dioxane (90%, v/v) as they mimic the extracellular matrix and membrane environment [34,35]. As shown in Fig 5, the spectrum obtained in water had a negative trough around 195 nm but was devoid of any alpha helical conformations. It is consistent with the negative minimum at around 195 nm (n→ π* shift) and the absence of positive band around 222 nm (π → π* shift). It indicates a random coil structure. The structure of the peptide was also analyzed in different concentrations of TFE. At 45% TFE (in water), there was an increase in negative ellipticity at around 210–220 nm along with positive ellipticity (+3000 deg cm^2^ dmol^-1^) at around 190–200 nm. Further increase of TFE concentration to 90%, led to a sharp increase in positive ellipticity (+8000 deg cm^2^ dmol^-1^) at around 190–200 nm and the negative band around 208 nm was clearly visible. Increment of TFE (45% to 90%, v/v) concomitantly decreased the 195 nm negative band with a crossover to have strong positive CD band with a negative double dichroic band at 208 nm with 220 nm shoulder. Further, the presence of a negative band at around 218 nm indicates the presence of β structure in the peptide. There was also a weak negative shoulder at around 222 nm. Inception of such CD features is due to induction of alpha helical population. It is clearly proved that polarity (biological environment) has significant effect on peptide backbone conformation and increment of TFE concentration or decrease in polarity led to increase in more ordered conformation and it was at the expense of random coil conformation in peptide. The CD spectra of kiss 1 peptide in 90% HFIP and 90% dioxane also showed similar features having mixed type of random, alpha helical, turn and beta being the major structure (Fig 6).

**Fig 7:**
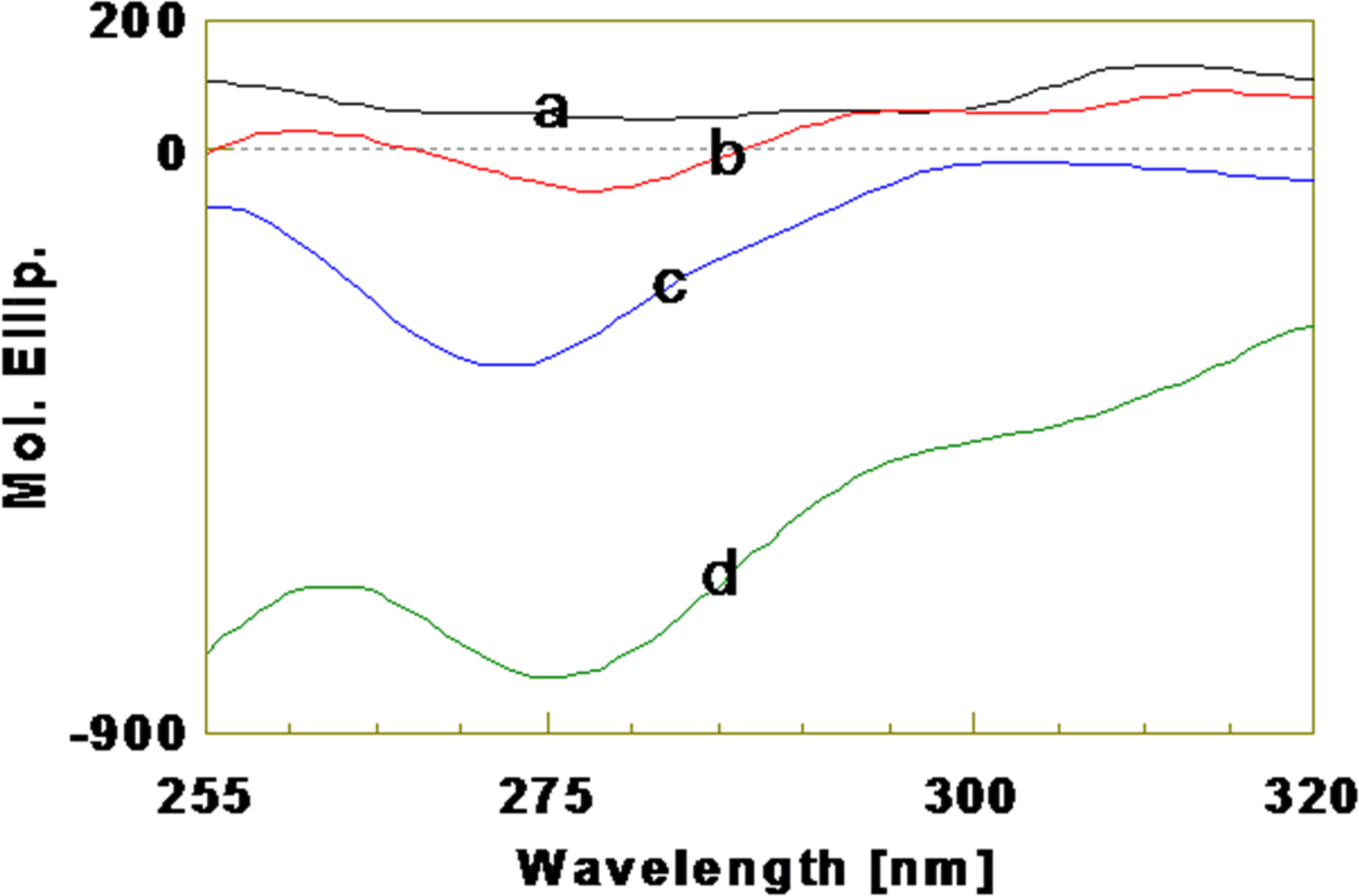
Near-UV CD spectra of tyrosine side chain residues of kiss 1 peptide in different environments. (a) CD spectrum in water. (b) CD spectrum in 45% TFE. (c) CD spectrum in 90% TFE. (d) CD spectrum in 90% dioxane.

**Fig 8:**
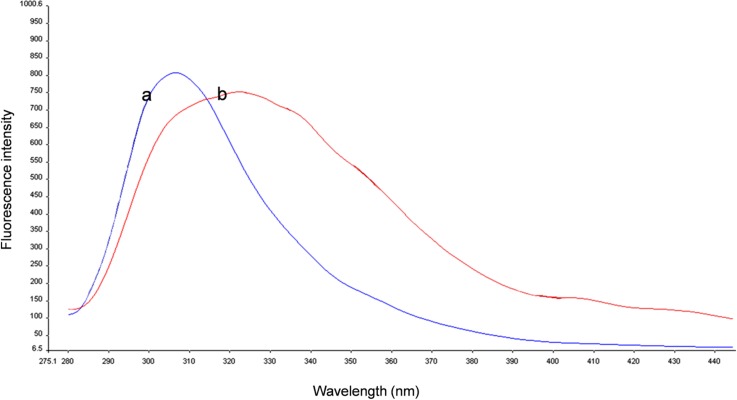
Fluorescence spectra of kiss 1 peptide. (a) Spectrum in water. (b) Spectrum in 90% TFE.

In order to study the tyrosine side chain conformational changes as a result of changes in polarity, CD spectra were recorded in near-UV region (255–320 nm). Fig 7 depicts CD spectra of aromatic tyrosyl side chain in kiss 1 peptide in different solvents used to simulate different low polarity environments that peptide may experience in biological systems. CD spectrum was recorded in water and have undefined feature suggestive of disordered conformation around tyrosine side chain. When the polarity of the environment was reduced using different concentrations of apolar solvent, TFE, there was inception of negative ellipticity with well defined negative trough at around 275 nm that concomitantly increased with increasing concentration of TFE. Further increase in negative ellipticity was observed when 90% dioxane was used. The results obtained from near-UV CD spectroscopy were further corroborated with fluorescence spectroscopy of kiss 1 peptide. The peptide showed different fluorescence maxima in water and TFE environment (Fig 8).

## Discussion

One of the fundamental principles of biology is that protein structure determines protein function. Based on this, small peptide mimetics of protein structures are emerging as new biomolecules which takes advantage of the chemical properties of the parent molecules.

In the present study, a 16 amino acid long putative mature bioactive peptide from preprokisspeptin 1 of golden mahseer was synthesized and characterized using an integrated (experimental and *in silico*) approach. The kiss 1 peptide was synthesized using Fmoc-chemistry, and its C-terminal was amidated as C-terminal amide group is crucial for all RFamide family proteins [30]. The peptide was eluted at around 60% acetonitrile with a retention time of 20.73 min which indicates that the peptide is slightly hydrophobic in nature. It is a basic peptide, and the iso-electric point is 10.4. The molecular mass characterized by MALDI-TOF-MS shows the unimolecular largest mass peak of 1977.58 Dalton, which conforms to the calculated mass of the peptide (1977.19 Dalton) and this validates the efficiency of synthesis.

In order to understand the conformation of the synthesized fish kiss 1 peptide, CD spectroscopy in different microenvironments was performed. In aqueous solution, CD spectrum of kiss 1 had a well defined negative extremum at 195 nm, which is a feature of random coil conformation [36]. Presence of random coil might provide flexibility for the adoption of ordered structures in different environments that occur in biological systems. In agreement, the peptide structures generated in PSI-PRED and GOR4 prediction tools also showed random coil to be the major conformational populations. With the addition of apolar solvent, i.e., TFE up to 45 and 90%, the CD spectrum showed interesting changes with the appearance of double dichroic negative band at nearly 208 and 220 nm, and a cross over strong positive maxima at 195 nm. Such features are attributed to the induction of alpha helical population in the peptide at the expense of random coil. These conformational changes in fish kiss 1 were discrete from previous observations in some mammalian kiss 1 counterparts such as human Kp10 in dodecylphosphocholine (DPC) micelles. Nuclear magnetic resonance (NMR) analysis of human Kp10 (in DPC micelles) showed that the region surrounding the residues tryptophan 3 to phenylalanine 10 contained several tight turn structures, but no helical conformation, with leucine 8 in the same hydrophobic cluster as phenylalanine 6 and 10 [37]. However, in sodium dodecyl sulfate (SDS) micelles, NMR studies of human Kp13 showed helical structure which suggests that the disruption of this conformation could be possible reason for the lower activity of improperly substituted compounds [38]. It is interesting to note that the conformation of human kisspeptin depends on the composition of membrane in which it is located. On the other hand, rat Kp10 mainly adopts a combination of helical and disordered conformations in diphenylcarbodiimide (DPCD) micelles [39]. Corresponding to the CD spectum, the 3D model of fish kiss 1 peptide also suggests the presence of a stretch of helical structure. The differential *in-silico* structures of fish kiss 1 peptide observed in PSIPRED, GOR and PEPFOLD concurs with a previous observation on the conformation of an antimicrobial peptide P2-Hp-1935 from skin secretions of frog, *Hypsiboas pulchellus* [40]. The peptide, P2-Hp-1935 had random coil conformations in PSIPRED and GOR tools as major population but no helical structure. However, the same peptide exhibited helical structure in 3D modelling as analysed by PEPFOLD and CD in TFE.

We also observed beta turn segment in the C-terminal of the fish kiss 1 peptide using CD spectroscopy and *in-silico* analysis, as reported in case of mammalian kisspeptin peptide, where turn structures were shown to be associated with biological activity [37]. Importance of the five C-terminal residues in receptor binding and activation has already been demonstrated in human kisspeptin analogs [41]. In structure-activity relationship studies of mammalian Kp-10 by different group of researchers, it was found that the nature as well as orientation of the side chains of five C-terminal residues are important for receptor binding and activation, whereas the N-terminal residues are more lenient towards substitution by L-alanine or enantiomer residues [38,41–45]. Moreover, we observed increase in the turn structures (6.8 to 15.5%), when shifting from aqueous to low polarity environment using dioxane. Our synthetic analog of golden mahseer kiss 1 exhibited disordered conformation in water but adopted ordered structure when exposed to membrane mimicking solvents like TFE, HFIP and dioxane. Specifically this peptide adopts a high proportion of β structure in different membrane mimetic environment due to the presence of aromatic amino acids corroborating with a recent study that reported ovine kisspeptin 14 adopts dominant β conformation in solvents such as TFE and HFIP [46]. Importance of aromatic residues, phenylalanine and tyrosine in agonistic activity was confirmed by alanine scanning experiment on rat Kp10 [39]. The chirality as well as aromatic properties of the C-terminal residues (arginine and phenylalanine or tyrosine) are also critical to human Kp10 activity because replacement of these amino acid residues by their D-enantiomers [41] or substitution by saturated-side-chain amino acids [38] significantly reduces their biological activity. Change in structural conformation of fish kiss 1 peptide in different solutions, from being random in aqueous to more ordered in biological environment mimetic solutions is similar to that of antimicrobial peptides (AMPs), which adopt a random coil structure in aqueous environment but changes to more ordered conformation while encountering bacterial membrane mimetic environment [47]. In fact, the fish kiss 1 peptide adopts more regular conformations, helical, turn and β-strand in membrane mimetic environment. Besides, the fish kiss 1 peptide had a significant random coil structure in both polar and apolar solvents. This random coil portion may favour the non-putative receptor binding for its multifunctional role, such as suppressor of cancer metastasis [48].

The near-UV CD spectroscopy that we carried out to examine the tertiary packing around aromatic residues of fish kiss 1 peptide and the kiss 1 peptide-membrane complex revealed a CD spectrum in water that had undefined features suggestive of disordered conformation around tyrosine side chain. Decreasing the polarity using different concentrations of apolar solvent (TFE) resulted in the inception of negative ellipticity with a well defined negative trough at around 275 nm, which was to increase concomitantly with increasing concentration of TFE. Likewise, the negative ellipticity was observed to increase further when the peptide was exposed to dioxane. Taken together, near-UV CD spectra of the peptide in water, TFE and dioxane are suggestive of different conformation for the tyrosine side chain. This was also corroborated by the fluorescence spectra of fish kiss 1 peptide in water and TFE which showed fluorescence maxima at different wavelengths. Tyrosine residues in the peptide may interact to form a hydrophobic cluster in aqueous environment which gets disrupted when secondary structure enhancer solvent like TFE was added to induce ordered helical population, resulting in exposure of the tyrosine side chains.

## Conclusion

In short, we synthesized and characterized a 16 amino acid long peptide derived from preprokisspeptin 1 of golden mahseer, and subjected it to solution conformational analysis. The peptide was found to adopt ordered helical, turn and β conformations in various membrane mimicking environments, which may favour the putative receptor binding of the peptide. Eventually, this peptide-receptor interaction may stimulate the secretion of GnRH at BPG axis and induce gonadal maturation. To the best of our knowledge, this is the first report on solution conformational study of a fish kiss 1 peptide. Further, the information generated in the present study can be utilized for designing highly potent bioactive synthetic peptide analogs which could improve the reproductive performances of commercially important fish species.
